# CT vs. bioluminescence: A comparison of imaging techniques for orthotopic prostate tumors in mice

**DOI:** 10.1371/journal.pone.0277239

**Published:** 2022-11-04

**Authors:** Molly S. Myers, Elizabeth A. Kosmacek, Arpita Chatterjee, Rebecca E. Oberley-Deegan

**Affiliations:** Department of Biochemistry and Molecular Biology, University of Nebraska Medical Center, Omaha, NE, United States of America; Stanford University, UNITED STATES

## Abstract

Prostate cancer is one of the most diagnosed cancers in men in the United States. In mouse models, orthotopic tumors are favored for their biological relevance and simulation of growth in a microenvironment akin to that found in humans. However, to monitor the disease course, animal models require consistent and noninvasive surveillance. *In vivo* bioluminescent imaging has become a mainstay imaging modality due to its flexibility and ease of use. However, with some orthotopic prostate tumor models, bioluminescence fails to describe disease progression due to optical scattering and signal attenuation. CT scanning, in addition to its utility in human cancer diagnosis and surveillance, can be applied to mouse models with improved results. However, CT imaging has poor definition when imaging soft tissues and is not routinely used in prostate cancer models. Using an orthotopic prostate cancer model, our results demonstrate that, when compared to bioluminescent imaging, CT imaging correlates more closely to orthotopic prostate tumor growth in mice. Based on the data from this study, we conclude that CT imaging can be used as an alternative to the more commonly used bioluminescent imaging for measuring orthotopic prostate cancer growth over time.

## Introduction

As the second leading cause of death in the United States, approximately 39.5% of people will be diagnosed with cancer at some point in their lifetime, highlighting the importance of developing novel diagnostics and therapeutics for this widespread disease [1]. Although research traditionally assays human cancer cells *in vitro*, mouse models allow for a more comprehensive study of pathogenesis inside a living subject [2]. Subcutaneous tumor models can be used to model tumor growth inside a living being, but orthotopically implanted tumors allow interaction with the relevant tumor microenvironment, as well as modeling natural metastasis [3].

One specific area of cancer research that benefits from orthotopic mouse models is prostate cancer. 1 in 8 men will be diagnosed with prostate cancer in their lifetime, and although many cases are indolent and prognosis is generally favorable, clinically aggressive prostate cancers are more likely to invade and metastasize regardless of treatment, resulting in worse outcomes [4]. Patients with distant disease have only a 31% five year survival rate, which is much lower when compared to the near-100% five year survival rate found in earlier detected, local disease [5]. Characterizing prostate cancer progression from primary to metastatic is vital to improving outcomes. Mouse models and orthotopic implantation of human cancer cells are especially important for studying prostate cancer, as it allows focused inquiry into the primary growth cascade that occurs in aggressive phenotypes [6]. Prostate cancer is also affected by its microenvironment; thus, studying the tumor within the confines of its natural anatomical position better mimics the human disease [7].

Unfortunately, monitoring orthotopic prostate tumor growth is difficult without sacrificing the animal. Imaging allows for a series of tumor measurements over time, providing valuable data on size and disease progression. Optical imaging modalities such as whole animal bioluminescent imaging are simple and widely used due to their specificity and versatility, as cells can either be transfected with genes for bioluminescent enzymes or tagged with a fluorescent probe [8]. One of the most widely used methods to monitor orthotopically implanted tumors uses cells transfected with luciferase, an enzyme that emits light when it interacts with its substrate, luciferin. The substrate is injected, allowing it to be imaged noninvasively, a technique which takes images and provides pixel intensities for a specific region of interest to estimate tumor growth [9]. However, any optical imaging modalities are subject to light scattering and light absorption. As such, optical imaging can be hindered by tissue depth and is generally limited to small animal rodent models [10].

The bioluminescent technique was developed, and its accuracy was reported, using subcutaneous tumor implants [11, 12]. While bioluminescence has shown correlation to actual tumor volume in orthotopic models, it is more variable [12, 13]. Accurately measuring tumors becomes challenging when characterizing orthotopic growth, particularly when tumors start to grow beyond normal anatomical scope, as they can grow to large sizes without impeding animal movement or viability. Further, larger tumor volumes can result in necrotic centers, alterations in blood supply causing hypoxia, and aberrant metabolism, all of which can lead to diminished luciferin concentrations and enzyme activity, decreasing fluorescent signals and falsely diminishing fluorescent intensity [14]. These problems have the likelihood to increase variability, which diminishes the utility of this technique to evaluate primary cancer growth and metastasis.

RM-1 cells, a mouse prostate carcinoma cell line, have mutated *ras* and *myc* oncogenes, resulting in androgen insensitivity and aggressivity [15]. RM-1 cells produce fast-growing, aggressive tumors when implanted in mice with a functioning immune system, providing a useful model for studying aggressive prostate cancer. LNCaP cells are an androgen sensitive human prostate cancer cell line well-known as a model for early prostate cancer progression [16]. Traditionally, long-term imaging of both hind flank and orthotopic prostate tumors is done using bioluminescent imaging due to its ease and convenience [17]. However, we were unable to transfect RM-1 cells with luciferase, which made bioluminescent imaging impossible. In addition, bioluminescent imaging was found to be inaccurate in measuring luciferase expressing LNCaP tumors.

Therefore, to monitor long-term tumor growth, we opted to use an alternate imaging method. Computed tomography (CT) imaging is used in humans to locate and characterize tumors, track invasion and metastasis, and plan therapeutic intervention and treatment [18]. In prostate cancer, CT is used for evaluating metastases, mostly by detecting enlarged lymph nodes, and for planning external beam radiation therapy to delineate potential healthy tissue involvement in patients’ treatment [19]. For small animals, CT imaging provides a viable modality to assess a primary tumor’s growth over time but is generally not employed in prostate cancer. CT imaging usually has poor contrast with soft tissues and is not used as a standard method to measure prostate tumor growth. We have found that we can use anatomical landmarks to reliably measure the prostate and prostate tumor. Thus, we hypothesize that CT tumor measurements correlate more closely to tumor size as compared to bioluminescent imaging in small animals with orthotopically implanted prostate tumors and that CT imaging can be used as an alternative method for measuring prostate tumor growth over time.

## Results

### Iodine contrast dye enhances CT imaging and identification of prostate

**Fig 1:**
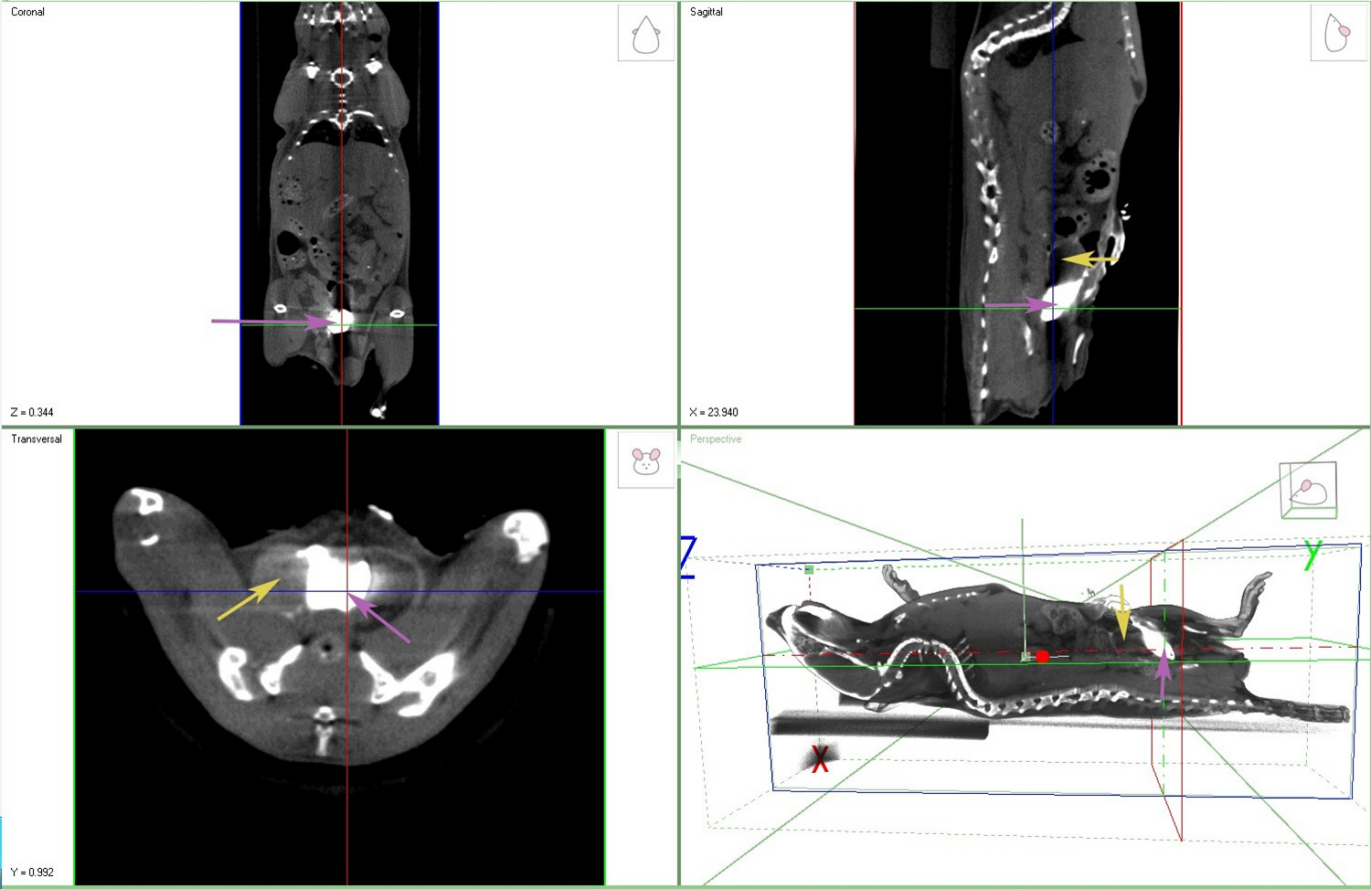
Iodine contrast agent used to identify mouse prostate. Organically bound iodine was directly injected into the prostate of a recently deceased mouse. A CT demonstrating high contrast between the prostate tissue and surrounding soft tissues. Purple arrows indicate the prostate in each view. Yellow arrows indicate the bladder in views where it is in plane. (n = 1).

Reading and interpreting CT scans requires knowledge of a subject’s normal anatomy, so we validated the ability to accurately identify and image the margins of a normal prostate using iodine contrast with CT imaging in a deceased mouse (Fig 1). This provided a frame of reference to locate the prostate’s position to other peritoneal structures such as the bladder and rectum that have pronounced contrast in CT imaging. The bladder is often filled with urine and the rectum/colon is filled with air providing distinct contrast as opposed to a solid tissue like the prostate. The organs and space surrounding the prostate are different densities so this region is uniquely arranged to allow for identification of these organs on CT despite being soft tissues.

### Anatomical geography is a reliable method to locate prostate on CT

**Fig 2:**
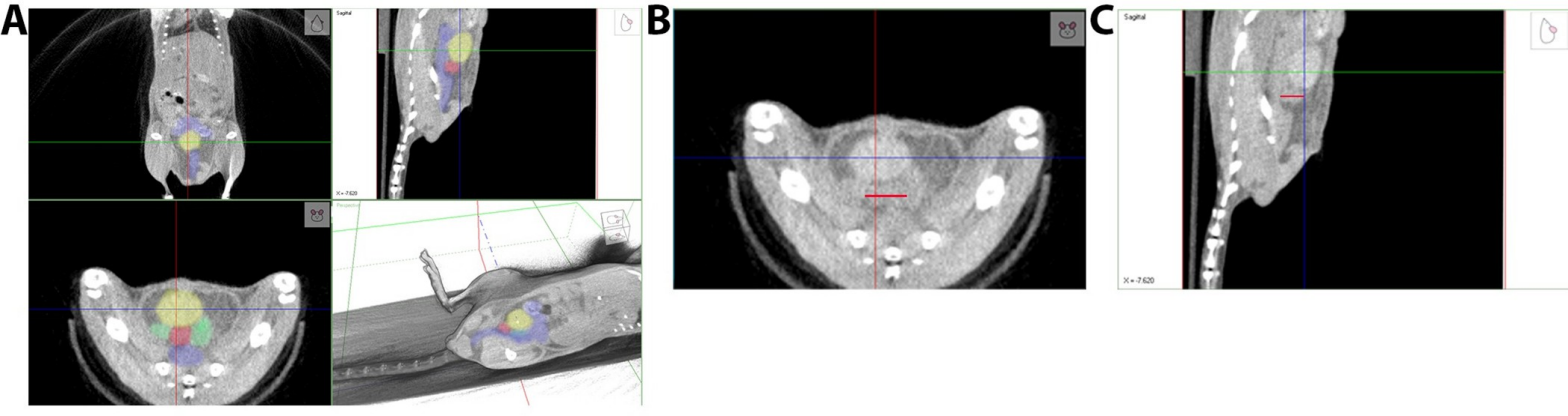
CT image of a normal mouse prostate and adjacent organs without image contrast agent. A non-contrast CT scan of a live mouse with a normal sized prostate. **A.** CT with labeled pelvic anatomy. The crosshairs are centered on the bladder. From the bottom left image, transverse view, the bladder (yellow) is above the prostate (red) and seminal vesicles (green), and below those structures is the colon (blue). **B.** Transverse view of the pelvis. The red line shows the transverse prostate boundaries. **C.** Sagittal view of the prostate. The red line shows the sagittal prostate boundaries. (This data is representative of 9 mice).

Once the prostate was located using a contrast agent, this method was applied in a live, breathing mouse after an operator was trained to find the prostate without the use of contrast. Although breathing causes imaging artifacts, we found that, especially when the mouse has a full bladder, the prostate was easily identified and was measurable within a few tenths of a millimeter (Fig 2). In Fig 2A, other anatomical landmarks are highlighted along with the prostate. The bladder is shown in yellow, prostate in red, seminal vesicles in green and the colon in blue in all three planes. In Fig 2B and 2C, the anatomical structural landmarks are unlabeled, and the prostate is denoted with the red line to highlight prostate dimensions in the different planes. We found the optimal conditions for CT imaging to be early morning due to mice eating and drinking during the dark cycle. In 6–8-week-old C57Bl/6J mice, normal prostate dimensions range between 4.6–5.3 mm in either dimension. For NU/J mice at 6–8 weeks of age, normal prostate dimensions range from 3.7–4.5 mm in either dimension.

### CT can accurately measure tumor growth over time

**Fig 3:**
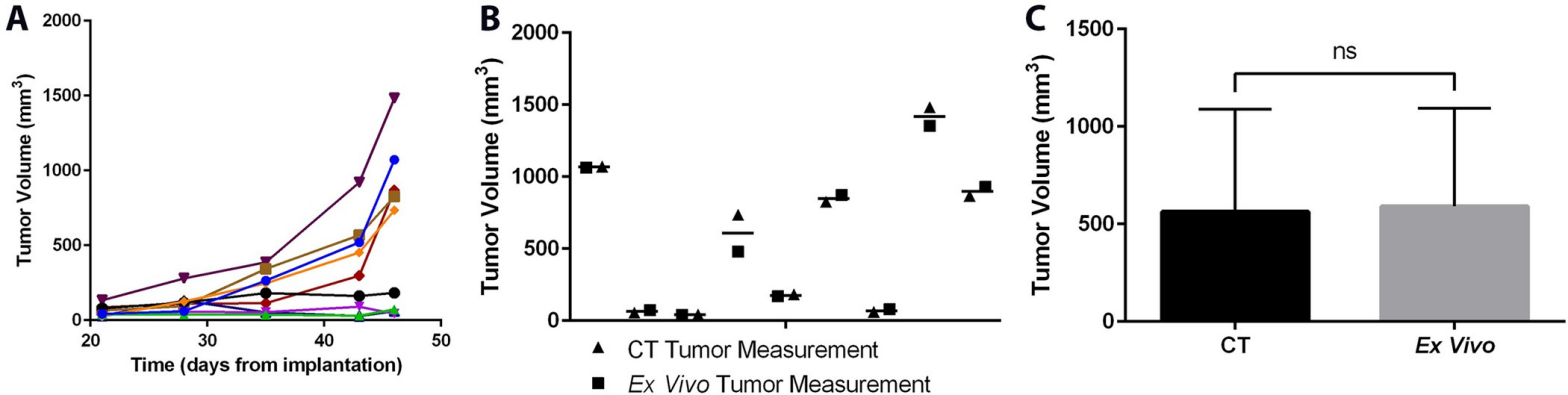
RM-1 tumor growth measured with CT as compared to *ex vivo* measurement. RM-1 tumors were orthotopically implanted into C57Bl/6J mice and allowed to grow for three weeks. **A.** Tumor growth measured by CT imaging weekly until 46 days post-implantation. **B.** The tumors were removed and measured *ex vivo* with calipers for comparison to the final CT calculated volume. **C.** Comparison of estimated tumor volume in CT and *ex vivo* caliper tumor measurement at 46 days implantation, n = 9 mice.

RM-1 cells, although ideal for studying prostate cancer growth and metastasis in immune competent mice, were not able to be stably transfected to express luciferase. Therefore, an imaging modality other than bioluminescence was required to construct a tumor growth curve. Starting at 21 days post implantation, animals were imaged using CT regularly until most had entered an exponential growth phase (Fig 3A), at which point the tumors were removed. The final CT, at 46 days post-implantation measurement was compared to gross measurement of the excised tumor using calipers (Fig 3B). For 8 of 9 animals, the measurements used to calculate the volume of the tumors differed by less than 1mm (average 0.15mm), resulting in estimated volumes that differed by less than 100mm^3^. In one animal, the tumor was overestimated in two dimensions by more than one millimeter, which resulted in an overestimated volume of 255 mm^3^. When comparing the tumor volumes for all 9 mice using CT vs excised *ex vivo* measurements, there was no significant difference in average calculated tumor volume using these two methods (Fig 3C). These data indicate that using CT imaging measurements were no different than external measurements made on excised tumors using calipers.

### CT provides more accurate tumor growth measurements as compared to bioluminescent imaging in a prostate orthotopic model

**Fig 4:**
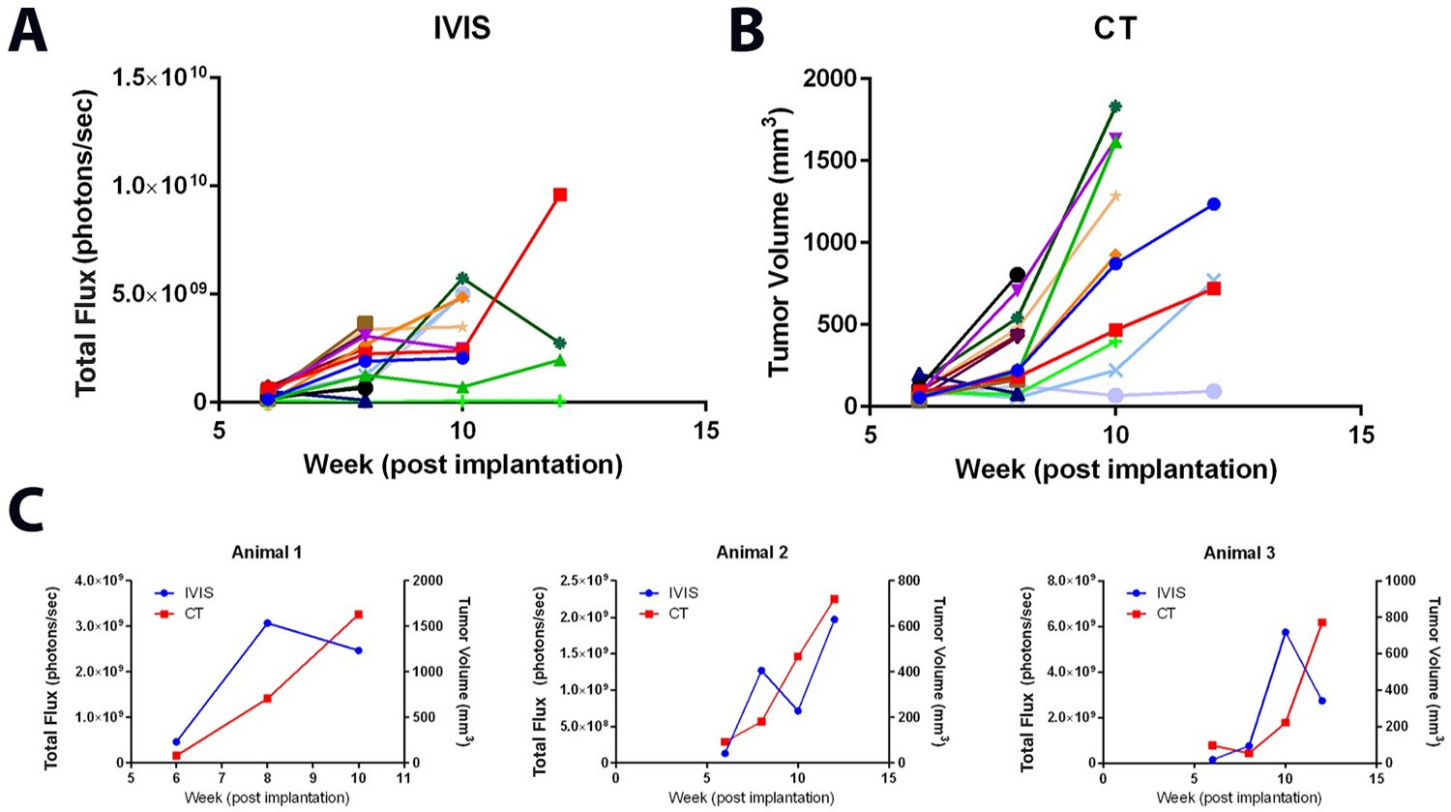
LNCaP orthotopic tumor growth estimated with bioluminescence or CT. LNCaP tumors growing in NU/J mice were measured with **A** IVIS or **B** CT. **C** Each graph represents a different mouse. Comparing the growth curves generated from the two imaging techniques, in several animals, it is common to observe typical exponential growth in the CT imaged tumors and abnormal increases and decreases in the IVIS generated growth curves, n = 20 mice.

To monitor the growth of tumors using optical imaging techniques, LNCaP cells were transduced to express the enzyme luciferase, which can be supplemented with the substrate d-luciferin to emit light (LNCaP-Luc). We then orthotopically implanted LNCaP-Luc prostate cancer cells into NU/J mice and tracked their growth using either bioluminescence imaging with IVIS (Fig 4A) or CT imaging (Fig 4B). Expected exponential growth is evident in almost all mice when tumors are measured by CT imaging (Fig 4B). In contrast, using bioluminescent imaging to estimate tumor growth over time resulted in erratic tumor growth curves (Fig 4A). A few representative tumor bearing mice from this cohort have been plotted to emphasize the difference of measurements between the two methods in Fig 4C.

**Fig 5:**
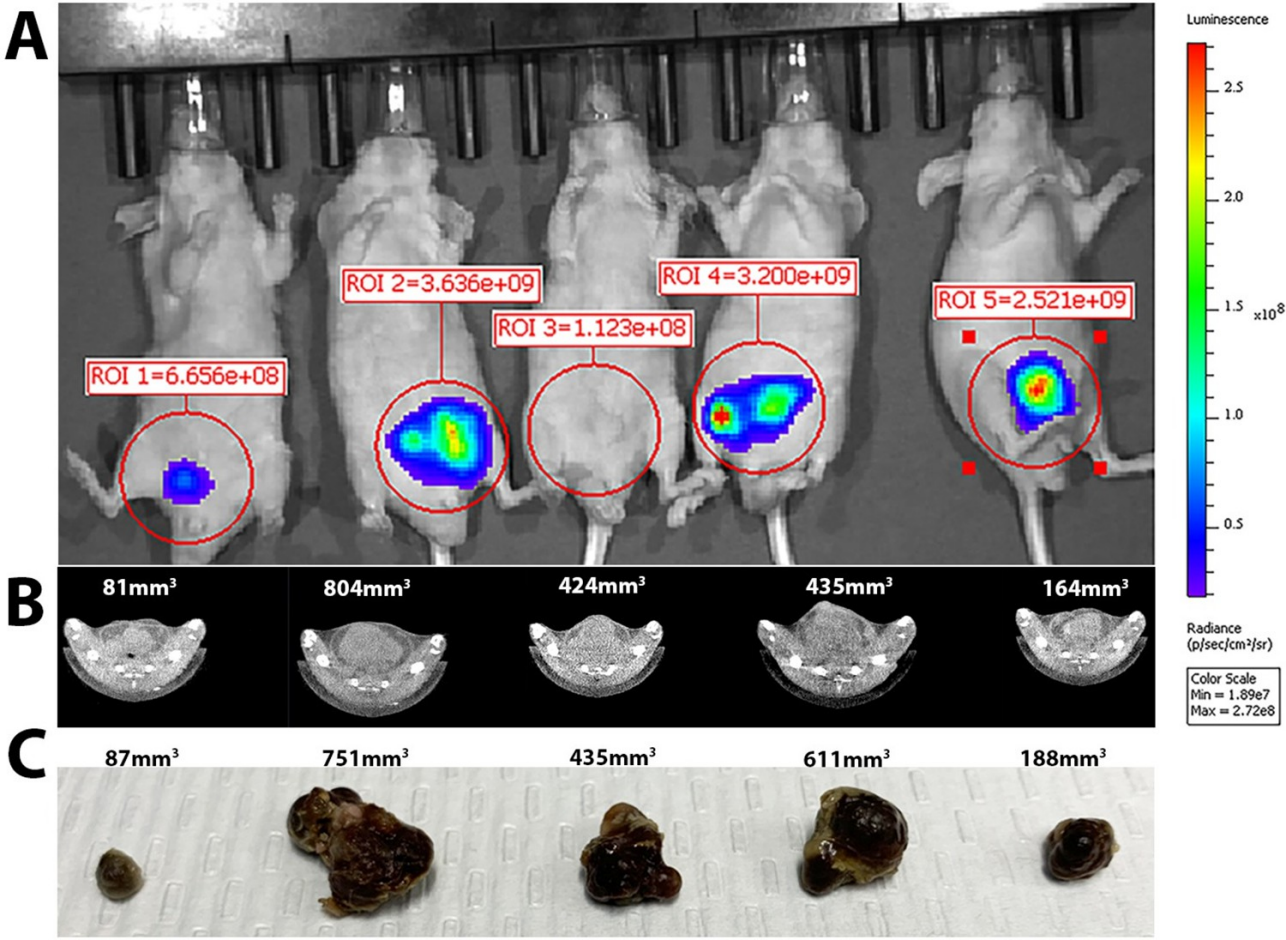
CT imaging more closely reflects *ex vivo* tumor size as compared to bioluminescence imaging. LNCaP prostate tumors at 8 weeks post-implantation. **A** Bioluminescent imaging used to measure orthotopic prostate tumors. **B** CT imaging used to measure the same orthotopic prostate tumor. **C.** Excised tumors measured with calipers for comparison at this 8-week time point, n = 5 mice.

To provide a direct visual comparison of bioluminescence imaging, CT, and *ex vivo* measurements, a representative comparison of each imaging modality is shown in Fig 5A–5C. Orthotopic LNCaP tumor measurements at 8 weeks post-implantation obtained from CT correspond more closely to the *ex vivo* measurements as compared to the bioluminescence method.

**Fig 6:**
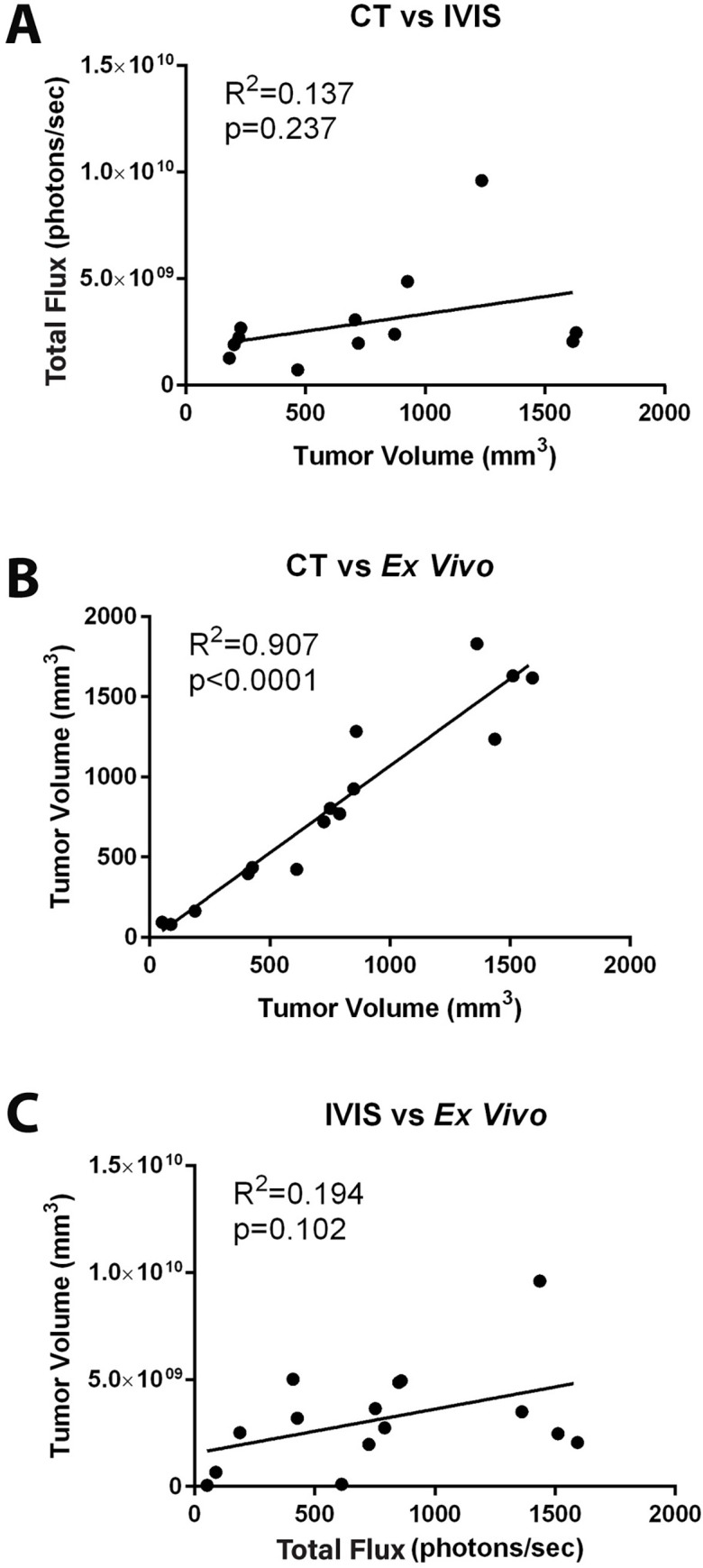
CT imaging significantly correlates with *ex vivo* tumor measurements. LNCaP tumors were removed at 8, 10-and 12 weeks post-implantation immediately following imaging. **A.** CT and IVIS bioluminescence imaging show no significant correlation to one another. **B.** CT and *ex vivo* tumor measurements are significantly correlated. **C.** IVIS bioluminescence imaging and *ex vivo* measurements have no significant correlation, n = 15 mice.

To validate each measurement over the course of the experiment, cages of animals were randomly selected for termination to remove the tumors at 8-, 10-, and 12-weeks post- implantation. Tumor measurements were verified externally by caliper. Prior to sacrifice the tumor size was measured via CT or bioluminescence imaging. We found no statistical correlation between CT measurements and bioluminescence imaging measurements (Fig 6A). CT imaging measurements were significantly correlated to caliper measurements taken upon removal of the tumor (Fig 6B). Bioluminescence imaging measurements did not correlate significantly with caliper measurements taken after tumor removal (Fig 6C). Thus, CT imaging of orthotopic Luc- LNCaP cells was a more accurate method to measure tumor growth over time as compared to bioluminescent imaging.

## Discussion

CT imaging more accurately correlates to *ex vivo* tumor size when measuring orthotopically implanted prostate tumors when compared with bioluminescent imaging. In fact, bioluminescent imaging measurements were highly variable in our LNCaP model. Bioluminescence has proven to be a reliable method for many tumor models, including orthotopic models, but there are certain models where bioluminescence does not accurately measure a growing tumor [13]. Previous studies have shown a bioluminescent plateau, where signal detects a mass but underestimates total burden as size increases [20, 21]. There are limitations to correlating total flux data to a growth curve, especially in internal organs, primarily due to optical scattering and interference. The farther bioluminescent rays travel through tissue, the more likely the signal will be lost. Since correlating bioluminescence data to size was first validated using flank tumors with minimal scatter and signal interference, it follows that the variability would diminish reliability and measurement accuracy. Indeed, this variable correlation has been found in some orthotopic tumor studies [13]. Additionally, signal detection requires either bioluminescent transfection or fluorescent tagging. In the case of luciferase transfection, the signal intensity depends on a variety of factors. A large tumor not only increases scattering, but adjusts to its microenvironment, resulting in alterations in blood supply, pH, and oxidative substrates, all key components that regulate oxidoreductase reactions, like luciferase [14]. Larger tumors may contain necrotic cores, aberrant blood supply, and adjustments in surrounding stroma that do not allow luciferin access, falsely diminishing signal and underestimating size [22, 23]. Finally, even if bioluminescent transfection or fluorescent tagging is possible, it may not be desirable. Primary human cancer and metastases are neither transfectable nor taggable, and a diagnostic animal model meant to mimic human physiology benefits from using clinically relevant, translatable imaging methods.

CT allows for a three-dimensional picture to be composed, providing volumetric data, without probes or tags. While bioluminescence data can detect a mass with variable correlation to its true size, a CT scan can be used to accurately measure the tumor volume. CT’s ability to detect metastasis, especially when combined with contrast or positron emission tomography, is more precise than bioluminescence by providing anatomical feedback to the extent of disease. Although our present study did not investigate metastases, machines such as micro-CTs, which provide high resolution CT images, have demonstrated competency when imaging lung and liver metastases in other cancer models, suggesting its utility when examining metastatic disease as well [24, 25]. Alternatively, MRI gives good soft tissue contrast, but can be costly and time consuming to perform [26]. 3D ultrasound is another alternative method to measure prostate cancer growth over time but takes substantial amount of training to adequately perform these measurements [27].

3D volume measurements would likely have provided a higher correlation with excised tumor measurements. Although we only used 2-dimensional approximation in CT, by scanning the length and width of the tumor, we were able to measure based on the largest section and found it sufficient to provide an R^2^ value of 0.9373 when compared to *ex vivo* tumor measurements. If quick, longitudinal imaging is the goal, such as it is with bioluminescent imaging, 2D approximations will adequately provide this information. The relative speed, ease of use, and the versatility of detecting bioluminescent and fluorescent signals all make bioluminescence an attractive option for quantifying tumor growth. IVIS can image five mice at once, which is fast and efficient when compared to CT’s ability to image one animal at a time. However, as bioluminescent signal varies, one animal with particularly intense signal can drown out animals with less intense signal, requiring the operator to reposition animals. As CT imaging is also relatively quick (five minutes per mouse), the logistical advantage of IVIS over CT diminishes. A major concern for using CT over bioluminescence is the need for ionizing radiation, which, in mice, could potentially cause off-target effects [28]. Using low doses of radiation to gather CT images could cause long-term off-target effects in mice. However, the dose required for sub- millimeter resolution imaging is less than 1 centigray, which is far below the recommended dose of 76–80 gray for definitive prostate radiotherapy in humans or 5–7 gray whole body irradiation to result in mouse lethality [29, 30]. These tumor bearing animals only live a month or two after irradiation, which will not provide them the time to potentially develop any of these side effects. Our data also supports that tumors are growing exponentially despite regular CT imaging, suggesting that repeated exposure to CT radiation is not sufficient to interfere with tumor growth studies.

Despite its comparable speed and superior accuracy, CT’s biggest drawback when compared to IVIS is that it requires prior anatomical knowledge, especially without the use of labeled probes. In humans, reading CT scans requires years of medical training, and mouse anatomy is similarly challenging to learn and understand. However, as we have demonstrated, with practice and the use of exploratory techniques such as performing an initial experiment using contrast agents or browsing existing anatomical scans, it is possible for researchers and technicians to reliably image and identify structures. Further, with focused scans and consistent models, such as those used in prostate cancer, the necessary scope of anatomical knowledge narrows. As CT is versatile and applies to many dimensions of cancer research, it is not only feasible to learn to read CT scans, but also advantageous, as it allows researchers to gather higher quality data. To our knowledge this is one of the first studies that demonstrate the ability to use CT to accurately measure orthotopic prostate growth over time.

To conclude, we compared tumor growth measurements using bioluminescence and CT to quantify orthotopic prostate tumors. Our *in vivo* studies demonstrated that bioluminescence growth measurements are highly variable and do not reflect true tumor growth in the LNCaP orthotopically implanted model. Conversely, CT scans are highly accurate and precise at estimating true tumor size and dimensionality over time in two orthotopic prostate cancer models. CT scanning is also preferable when bioluminescent transfection or fluorescent tagging is not possible, like in the case of the RM-1 cell line. As CT scanning has become commonplace in detecting cancer clinically, adapting experimental models to reflect clinical approaches are important, especially when the alternatives maybe less precise.

## Materials and methods

### Cell culture

RM-1 cells were obtained from Dr. Leah Cook (Department of Pathology and Microbiology, UNMC). The RM-1 cells were maintained in RPMI-1640 media supplemented with 10% FBS and 1% penicillin/streptomycin in a humidified incubator at 37°C, 95% air and 5% CO2. LNCaP cells were purchased from ATCC (clone FGC cat. CRL-1740) and transduced with lentivirus expressing firefly luciferase (Genecopeia). LNCaP-Luc cells were maintained in RPMI-1640 media supplemented with 5% FBS and 1% penicillin/streptomycin in a humidified incubator at 37°C, 95% air and 5% CO2. Cells were tested regularly for mycoplasma contamination.

### Ethical approval of the study

This study was approved and performed under the institutional animal care and use committee at UNMC (20-019-03-FC). This study was carried out in strict accordance with the recommendations of the Guide for the Care and Use of Laboratory Animals of the National Institutes of Health. Mice were sacrificed using CO2 exposure followed by cervical dislocation. Isoflurane was used for anesthesia for surgery and imaging of mice. Animals were treated with buprenorphine as an analgesic after surgery. Animals were euthanized immediately upon displaying signs of reduced ambulatory function or distress.

### Experimental animals

6 to 8-week-old male C57BL/6J mice or athymic NU/J (Jackson Laboratories, Bar Harbor, ME) were used in this study. The mice were housed at the University of Nebraska Medical Center (UNMC), exposed to a 12 h light/dark cycle, and fed and watered *ad libitum*. For the contrast study, one mouse was used. For the RM1 CT experiment, nine mice were used. For the LnCAP CT vs. bioluminescence experiment, twenty mice were used.

### Orthotopic implantation of tumor cells

Mice were anesthetized by continuous flow of 2–3% isoflurane with 1% oxygen using a mouse anesthesia machine. The lower abdomen was cleaned with alcohol and iodine and a one cm midline incision was made. To expose the prostate gland, the bladder was gently retracted. RM- 1 cells (in C57Bl/6J) or LNCaP-Luc cells (in NU/J) were injected into the dorsal prostatic lobe in a total volume of 50 μL containing 2.0 × 10^6^ cells in 50% Matrigel using a 30-gauge needle. The peritoneal muscle tissue was closed using absorbable catgut sutures (cat. 563B, Surgical Specialties, Tijuana, Mexico) and the skin was closed with wound clips (cat. 1111C15, Thomas Scientific, Swedesboro, NJ, USA). Buprenorphine (0.1 mg/kg, Reckitt Benckiser Healthcare, UK Ltd., Hull, UK) was administrated by intraperitoneal injection just after the surgery followed by three doses at 6, 24, and 48 h post-surgery as an analgesic. Sterile surgical procedures were maintained for the entire process. Wound clips were removed after ten days, and animals were monitored daily for re-opening of the incision site, infection, or distress.

### CT imaging

Three weeks (RM-1) or six weeks (LNCaP-Luc) post implantation, and weekly thereafter, animals were CT imaged to monitor tumor growth. Animals were anesthetized by continuous flow of 2.7% isoflurane with oxygen. Cone beam CT images were acquired with the small animal radiation research platform, SARRP (XSTRAHL, Suwanee, GA). The animals are placed on a bed that rotates 360° between a stationary x-ray source (providing 60kVp, 0.8mA continuously) and a flat panel amorphous silicon detector. Cone beam CT images were acquired at a 0.6 × 0.6 × 0.6 mm^3^ voxel resolution.

For training purposes, a preliminary contrast-enhanced imaging study was performed on a single euthanized mouse to clearly identify the prostate. For contrast imaging, a euthanized mouse’s prostate was exposed with a three cm midline incision. The peritoneum was disturbed but internal organs were left unmanipulated to preserve their anatomical location. Fifty microliters of Isovue-370 (Bracco Diagnostics, Singen, Germany), an organically bound iodine contrast agent, was injected directly into the prostate lobes. The peritoneum and muscle layers were gently sutured to restore any displaced organs. A CT was acquired immediately to so that minimal leakage of iodine would occur to distort the visualization of the prostate.

The prostate is connected directly to the bladder and seminal vesicles and lays on top of the colon when a mouse is in the supine position. These geographical landmarks (bladder, the seminal vesicles and colon) provide enough contrast to identify the prostate on all three planes of a CT scan. A CT operator was trained on both images with contrast agent and without contrast agent to reliably measure the prostate before tumor implantation. Once the tumor begins growing the prostate tumor is more easily identified and measured. Prostates of healthy animals were dissected and measured, and the volumes were calculated and compared to CT images to validate the accuracy of the CT measurements. The operator was trained on ten mice.

### Tumor volume calculation

For both CT and *ex vivo* measurements with calipers, tumors were measured along two perpendicular axes usually in the transverse and sagittal (dorsal to ventral) planes. In CT imaging, the coronal plane was traversed to ensure the largest slice of the tumor was measured. The longest measurement was assigned to length (l) and the shorter measurement assigned to width (w). The volume was then estimated with the equation V = w^2^*l/2 and is expressed in units of mm^3^.

### Bioluminescent imaging

D-Luciferin potassium salt (100 mg/kg, PerkinElmer, cat. 122799, Waltham, MA, USA) was dissolved in PBS and then sterile filtered. LNCaP-Luc tumor bearing mice were injected intraperitoneally 15 minutes prior to imaging based on previous imaging studies (data not shown). Mice were anesthetized using continuous flow of 2.5% isoflurane with oxygen and placed in the Xenogen IVIS Spectrum bioluminescence imaging system (PerkinElmer, MA, USA). Luminescence was acquired for 1 second with the maximum field of view (FOV) of 24.5 cm, a bin size of 8 and lens aperture set to f1 (fully open for maximal photon collection). Images were analyzed using Living Image 4.5.1 software (Caliper Life Sciences, Hopkinton, MA, USA). Regions of interest (ROI) were determined visually to include the entire area demonstrating light output, and total flux was reported as a measurement of photons/sec.

### Statistical analysis

To compare CT to *ex vivo* caliper measurements, a student’s t-test was performed. We performed linear regression analysis and used Pearson correlation coefficients to evaluate the relationship between tumor growth measured by CT, bioluminescence imaging, and *ex vivo* measurements pairwise. Statistical significance was defined as p <0.05. All analysis was performed with Graph Pad Prism.

## References

[1] Institute NC. Cancer Stat Facts: Prostate Cancer 2021.

[2] Killion JJ, Radinsky R, Fidler IJ. Orthotopic models are necessary to predict therapy oftransplantable tumors in mice. Cancer and Metastasis Reviews. 1998;17(3):279–84. doi: 10.1023/a:1006140513233 10352881

[3] Talmadge JE, Singh RK, Fidler IJ, Raz A. Murine Models to Evaluate Novel and Conventional Therapeutic Strategies for Cancer. The American Journal of Pathology. 2007;170(3):793–804. doi: 10.2353/ajpath.2007.060929 17322365 PMC1864878

[4] Liu R, Yang K, Meng C, Zhang Z, Xu Y. Vasculogenic mimicry is a marker of poor prognosis in prostate cancer. Cancer Biology & Therapy. 2012;13(7):527–33. doi: 10.4161/cbt.19602 22407030

[5] Siegel RL, Miller KD, Fuchs HE, Jemal A. Cancer statistics, 2022. CA: A Cancer Journal for Clinicians. 2022;72(1):7–33. 35020204 10.3322/caac.21708

[6] Pavese J, Ogden IM, Bergan RC. An orthotopic murine model of human prostate cancer metastasis. J Vis Exp. 2013(79):e50873–e. doi: 10.3791/50873 24084571 PMC3814297

[7] Zhang W, Fan W, Rachagani S, Zhou Z, Lele SM, Batra SK, et al. Comparative Study of Subcutaneous and Orthotopic Mouse Models of Prostate Cancer: Vascular Perfusion, Vasculature Density, Hypoxic Burden and BB2r-Targeting Efficacy. Scientific Reports. 2019;9(1). doi: 10.1038/s41598-019-47308-z 31366895 PMC6668441

[8] Baker M. The whole picture. Nature. 2010;463(7283):977–9. 20164931 10.1038/463977a

[9] Zinn KR, Chaudhuri TR, Szafran AA, O’Quinn D, Weaver C, Dugger K, et al. Noninvasive bioluminescence imaging in small animals. ILAR J. 2008;49(1):103–15. doi: 10.1093/ilar.49.1.103 18172337 PMC2614121

[10] Close DM, Xu T, Sayler GS, Ripp S. In Vivo Bioluminescent Imaging (BLI): Noninvasive Visualization and Interrogation of Biological Processes in Living Animals. Sensors. 2010;11(1):180–206. doi: 10.3390/s110100180 22346573 PMC3274065

[11] Rice B, Cable M, Nelson M. In vivo imaging of light-emitting probes. Journal of Biomedical Optics. 2001;6(4). doi: 10.1117/1.1413210 11728202

[12] Tseng J, Vasquez K, Peterson J, Hopkinton M. Optical imaging on the IVIS SpectrumCT system: general and technical considerations for 2D and 3D imaging. Technical note, pre-clinical in vivo imaging. PerkinElmer. Inc, Waltham, MA. 2015.

[13] Shen YT, Asthana R, Peeters C, Allen C, Deangelis C, Piquette-Miller M. Potential Limitations of Bioluminescent Xenograft Mouse Models: A Systematic Review. Journal of Pharmacy & Pharmaceutical Sciences. 2020;23:177–99. doi: 10.18433/jpps30870 32407285

[14] Inoue Y, Tojo A, Sekine R, Soda Y, Kobayashi S, Nomura A, et al. In vitro validation of bioluminescent monitoring of disease progression and therapeutic response in leukaemia model animals. European Journal of Nuclear Medicine and Molecular Imaging. 2006;33(5):557–65. doi: 10.1007/s00259-005-0048-4 16501974

[15] Baley KY P.A., Qian W., Sehgal I., Thompson T.C. Progression to androgen insensitivity in a novelin vitro mouse model for prostate cancer. The Journal of Steroid Biochemistry and Molecular Biology. 1995;52(5):403–13. doi: 10.1016/0960-0760(95)00001-g 7538321

[16] Horoszewicz JS, Leong SS, Kawinski E, Karr JP, Rosenthal H, Chu TM, et al. LNCaP model of human prostatic carcinoma. Cancer Res. 1983;43(4):1809–18. 6831420

[17] Koo V, Hamilton PW, Williamson K. Non-invasive in vivo imaging in small animal research. Cell Oncol. 2006;28(4):127–39. doi: 10.1155/2006/245619 16988468 PMC4617494

[18] Bar-Shalom R, Yefremov N, Guralnik L, Gaitini D, Frenkel A, Kuten A, et al. Clinical Performance of PET/CT in Evaluation of Cancer: Additional Value for Diagnostic Imaging and Patient Management. Journal of Nuclear Medicine. 2003;44(8):1200–9. 12902408

[19] Calais J, Cao M, Nickols NG. The Utility of PET/CT in the Planning of External Radiation Therapy for Prostate Cancer. J Nucl Med. 2018;59(4):557–67. doi: 10.2967/jnumed.117.196444 29301928 PMC6910632

[20] Scatena CD, Hepner MA, Oei YA, Dusich JM, Yu S-F, Purchio T, et al. Imaging of bioluminescent LNCaP-luc-M6 Tumors: A new animal model for the study of metastatic human prostate cancer. The Prostate. 2004;59(3):292–303. doi: 10.1002/pros.20003 15042605

[21] Sarraf-Yazdi S, Mi J, Dewhirst MW, Clary BM. Use of in vivo bioluminescence imaging to predict hepatic tumor burden in mice. J Surg Res. 2004;120(2):249–55. doi: 10.1016/j.jss.2004.03.013 15234220

[22] Forster J, Harriss-Phillips W, Douglass M, Bezak E. A review of the development of tumor vasculature and its effects on the tumor microenvironment. Hypoxia. 2017;Volume 5:21–32. doi: 10.2147/HP.S133231 28443291 PMC5395278

[23] Liu Z-G, Jiao D. Necroptosis, tumor necrosis and tumorigenesis. Cell Stress. 2020;4(1):1–8. 10.15698/cst2020.01.208 PMC6946014 31922095

[24] Li XF, Zanzonico P, Ling CC, O’Donoghue J. Visualization of experimental lung and bone metastases in live nude mice by X-ray micro-computed tomography. Technol Cancer Res Treat. 2006;5(2):147–55. 16551134

[25] Boll H, Nittka S, Doyon F, Neumaier M, Marx A, Kramer M, et al. Micro-CT Based Experimental Liver Imaging Using a Nanoparticulate Contrast Agent: A Longitudinal Study in Mice. PLOS ONE. 2011;6(9):e25692. doi: 10.1371/journal.pone.0025692 21984939 PMC3184160

[26] Ni J, Bongers A, Chamoli U, Bucci J, Graham P, Li Y. In Vivo 3D MRI Measurement of Tumour Volume in an Orthotopic Mouse Model of Prostate Cancer. Cancer Control. 2019;26(1):107327481984659. 10.1177/1073274819846590 PMC6488786 31032634

[27] Ni J, Cozzi P, Hung TT, Hao J, Graham P, Li Y. Monitoring Prostate Tumor Growth in an Orthotopic Mouse Model Using Three-Dimensional Ultrasound Imaging Technique. Transl Oncol. 2016;9(1):41–5. doi: 10.1016/j.tranon.2015.11.011 26947880 PMC4800064

[28] Meganck JA, Liu B. Dosimetry in Micro-computed Tomography: a Review of the Measurement Methods, Impacts, and Characterization of the Quantum GX Imaging System. Molecular Imaging and Biology. 2017;19(4):499–511. doi: 10.1007/s11307-016-1026-x 27957647 PMC5498628

[29] Wong J, Armour E, Kazanzides P, Iordachita I, Tryggestad E, Deng H, et al. High-Resolution, Small Animal Radiation Research Platform With X-Ray Tomographic Guidance Capabilities. International Journal of Radiation Oncology*Biology*Physics. 2008;71(5):1591–9. doi: 10.1016/j.ijrobp.2008.04.025 18640502 PMC2605655

[30] Li G, Li Y, Wang J, Gao X, Zhong Q, He L, et al. Guidelines for radiotherapy of prostate cancer (2020 edition). Precision Radiation Oncology. 2021;5(3):160–82.

